# AGE AND COHORT TRENDS OF RACIAL/ETHNIC DIFFERENCE IN RELIGIOUS PARTICIPATION AMONG MIDDLE-AGED AND OLDER AMERICANS

**DOI:** 10.1093/geroni/igad104.1298

**Published:** 2023-12-21

**Authors:** Jingwen Liu

**Affiliations:** University of Maryland, College Park, College Park, Maryland, United States

## Abstract

Religious involvement is an important way of maintaining social connectedness for older Americans. While large quantities of studies have explored age and cohort effects of religious participation separately, less is known about racial/ethnic disparities. Applying growth curve modeling to the 2004-2020 waves of the Health and Retirement Study (N=134,734 person-years), the current study examines how religious attendance changes across the life course and among recent birth cohorts, as well as how exposure to immigration policy regimes (IPR) shapes Hispanic immigrants’ religious participation behaviors. Results suggest an overall reversed U-shape age trajectory with lower average levels and faster declines among more recent cohorts. Compared to White older adults, Black and Hispanic populations attend religious activities more frequently, but their attendance declines at faster rates in later life, leading to minimal White-Nonwhite differences in the oldest ages. However, Hispanic immigrants display significantly less decline in religious participation among more recent cohorts relative to Whites, although no similar trend is found between White and Black/native Hispanic older adults. Further analyses of Mexican Hispanic immigrants suggest that longer exposure to restricted IPR (since the Immigration Reform and Control Act of 1986, IRCA) is associated with increased religious attendance, but less restricted IPR (between 1964 and 1985) is associated with decreased religious participation net of age and cohort effects. These findings emphasize the importance of understanding the civic engagement experiences of Hispanic immigrants against the context of immigration policy regimes.

